# Effects of cigarette smoke on *Haemophilus influenzae*-induced otitis media in a rat model

**DOI:** 10.1038/s41598-021-99367-w

**Published:** 2021-10-05

**Authors:** Sung-Won Choi, Sunmi Choi, Eun-Jin Kang, Hyun Min Lee, Se-Joon Oh, Il-Woo Lee, Hwan Ho Lee, Soo-Keun Kong

**Affiliations:** 1 Department of Otorhinolaryngology and Pusan National University School of Medicine, Biomedical Research Institute, Pusan National University Hospital, Gudeok-ro 179, Seo-Gu, Busan, 49241 Republic of Korea; 2 Department of Otorhinolaryngology and Pusan National University School of Medicine, Biomedical Research Institute, Pusan National University Yangsan Hospital, Yangsan, Republic of Korea; 3 Department of Otolaryngology and Kosin University College of Medicine, Kosin University Hospital, Busan, Republic of Korea

**Keywords:** Diseases, Infectious diseases, Respiratory tract diseases, Infection, Inflammation

## Abstract

Exposure to cigarette smoke (CS) is a factor that could delay or worsen the recovery of otitis media (OM) by causing inflammatory swelling of the Eustachian tube (ET). However, despite the suggested relationship, little is known about the association between OM and CS. Therefore, we aimed to evaluate the effects of CS on the development, progression, and recovery of OM, as well as the histological and molecular changes caused by CS exposure, by using a rat model of OM infected with non-typeable *Haemophilus influenzae* (NTHi). Eighty Sprague–Dawley rats with normal middle ears (MEs) were divided into four groups (n = 20 rats/group): control, CS, OM, and CS + OM. The CS and CS + OM groups were exposed to CS for 2 weeks. The inflammatory reaction to NTHi was more intense and lasted longer in the CS + OM group than in the other groups. Goblet cell proliferation and mucus secretion in the ET were more significant in the CS and CS + OM groups than in the other groups. These findings suggested that because CS directly affects the ET and ME mucosa, bacterial OM can become more severe and may resolve more slowly in the presence of CS exposure rather than in its absence.

## Introduction

Otitis media (OM) is one of the most common bacterial diseases associated with infancy and childhood^1–3^. Approximately 80% of children under 3 years old develop OM at least once^1–3^. OM often necessitates antibiotic treatments and surgical procedures during infancy and childhood^2^, because improper treatment can lead to complications such as meningitis, mastoiditis, and brain abscess^3^. OM is also a leading cause of childhood hearing loss and associated delays in language development, if not treated properly^1^. Although OM is a multifactorial disease involving Eustachian tube (ET) dysfunction, allergy, upper respiratory infection, and various environmental factors, the primary cause is bacterial infection of the middle ear (ME) mainly by *Streptococcus pneumoniae*, non-typeable *Haemophilus influenzae* (NTHi), or *Moraxella catarrhalis*^4^.

A hallmark of OM is hyperplasia and proliferative response of the mucosa lining the ME^5–8^. The ME mucosa transforms from a monolayer of simple squamous epithelium to a full-thickness pseudostratified columnar respiratory epithelium containing secretory and ciliated cells within a few days of developing OM, as seen in both animal models and humans^4–9^. The hyperplasia and proliferative changes contribute to the pathogenesis and symptoms of OM through the production of mucus and other bioactive secretions by various recruited inflammatory cells, as well as through ME cavity volume reduction^4^.

Exposure to cigarette smoke (CS) has long been identified as a major risk factor for airway diseases, including OM, emphysema, chronic obstructive pulmonary disease, allergies, and malignancy^10–13^. Clinically, CS is associated with an increased risk of developing chronic suppurative OM^14,15^. Smokers with this condition have high rates of active otorrhoea and aggressive cholesteatoma with further complications, including exposed dura, exposed facial nerve, and labyrinthine fistulas^16^. However, the mechanism by which CS causes or aggravates OM has not yet been fully understood. Similarly, it remains unclear whether OM related to CS has a different reaction to bacterial infections than does OM related to other etiologic factors. In animal models, CS directly affects the ET and ME mucosa during both short-term (1–8 weeks) and long-term (4–6 months) exposures by causing histologic changes involving goblet cell proliferation and excessive mucus secretion^17,18^. Thus, CS can be considered a factor that delays or worsens the recovery of OM by causing inflammatory swelling of the ET. Despite the suggested relationship between CS and the ET, little is known about the association between OM and CS, and few studies have investigated the effects of CS on the progression and recovery of OM.

In this study, we aimed to evaluate the effects of CS on the development, progression, and recovery of OM, as well as the histological and molecular changes caused by CS exposure, by using a rat model of OM infected with NTHi.

## Results

### In vivo studies

#### Induction of OM

**Figure 1:**
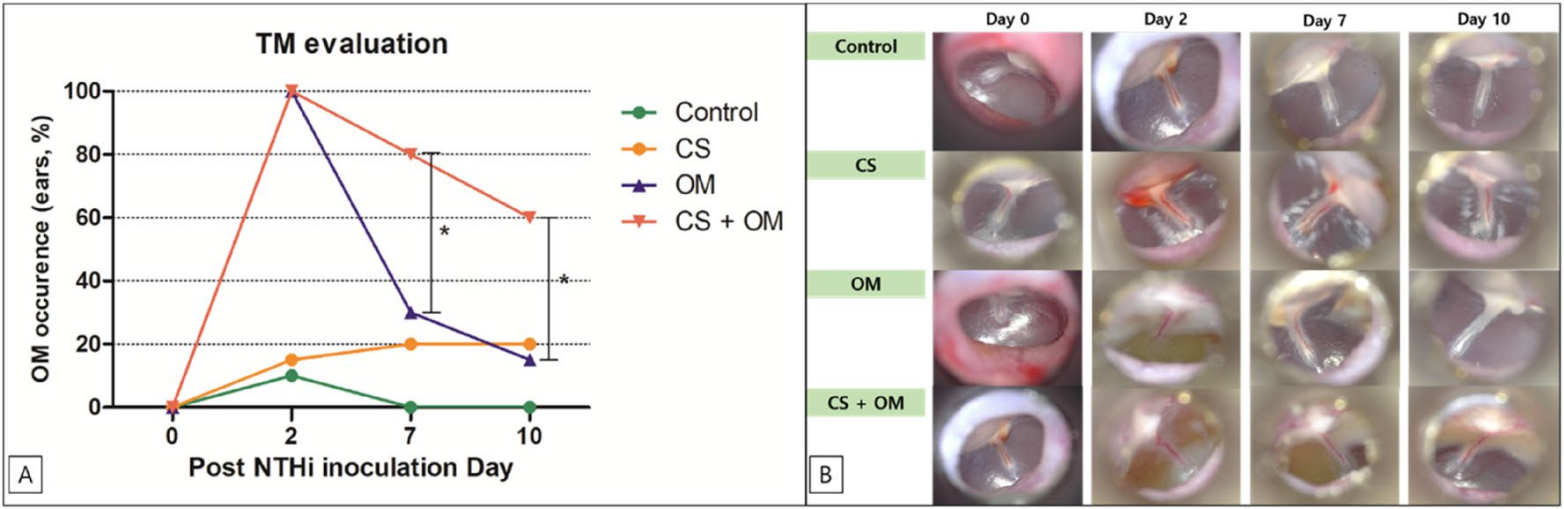
Induction of otitis media (OM). (**A**) The otitis media + cigarette smoke (CS + OM) group shows higher induction of OM than does the OM group on days 7 (P = 0.011) and 10 (P = 0.006) after inoculation. (**B**) Otomicroscopic examination findings of the tympanic membrane.

All the rats in the control group and experimental subgroups were alive during the research period and showed normal tympanic membranes before NTHi or phosphate-buffered saline (PBS) inoculation. The tympanic membrane was evaluated at each time point after NTHi or PBS inoculation (0, 2, 7, 10 days). On day 2, all the ears showed OM in both the OM (100%) and CS + OM groups (100%). Several ears in the CS (15%) and control groups (10%) also showed OM on day 2. On day 7, OM was observed in 80% of ears in the CS + OM group and 30% in the OM group. On day 10, 60% of ears in the CS + OM group still had OM, while only 15% of those in the OM group had OM. The induction of OM differed significantly among the four groups on days 2 (P < 0.001), 7 (P < 0.001), and 10 (P = 0.045) (Fig. 1). The CS + OM group showed higher induction of OM than did the OM group on days 7 (P = 0.011) and 10 (P = 0.006) (Fig. 1).

#### ME mucosal thickness

**Figure 2:**
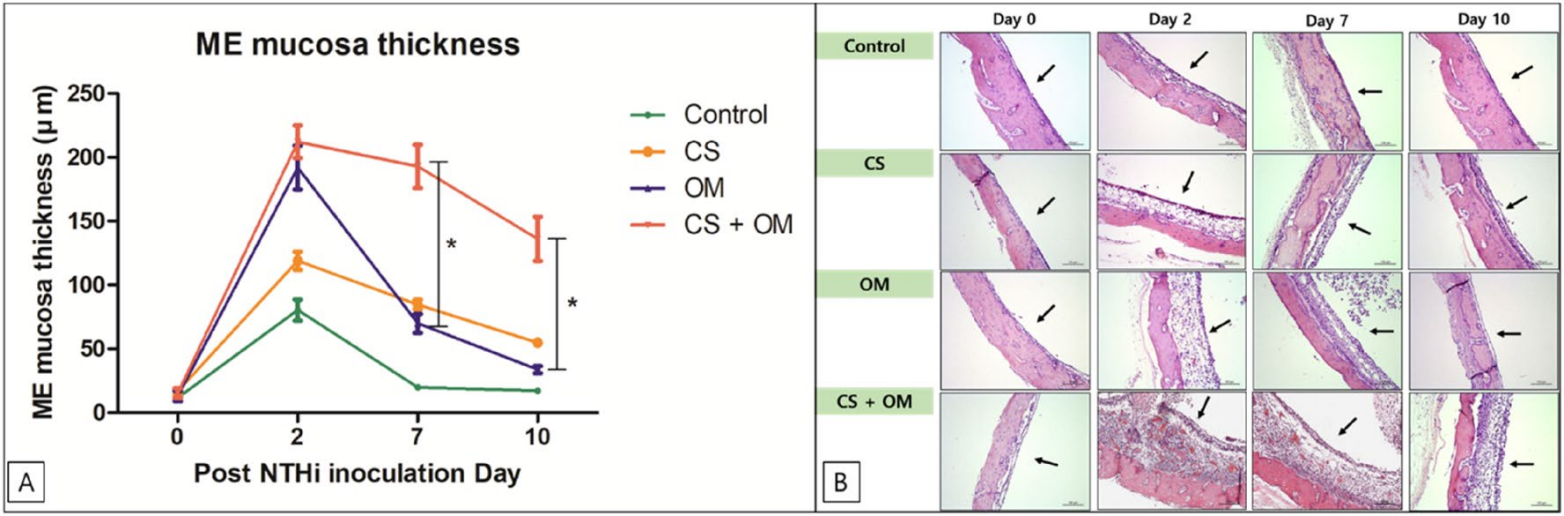
(**A**) Measurement of the middle ear (ME) mucosal thickness. (**B**) The ME mucosa is stained using hematoxylin and eosin (× 200 magnification).

ME mucosal thickness before inoculation was 16.88 ± 2.42 μm in the CS group and 15.52 ± 3.77 μm in the CS + OM group. Although not statistically significant, both thicknesses were greater than those in the control (11.67 ± 1.34 μm) and OM groups (13.07 ± 4.07 μm). ME mucosal thickness of all groups increased after PBS or NTHi inoculation. ME mucosal thickness showed a greater increase in the OM and CS + OM groups than in the control and CS groups at day 2 (P = 0.045). ME mucosal thickness was significantly greater in the CS + OM group than in the other groups on days 7 (P < 0.001) and 10 (P < 0.001) (Fig. 2). The OM group showed the peak thickness on day 2, and it decreased steeply thereafter, whereas the CS + OM and CS groups showed a gradual decrease in thickness until day 10 (Fig. 2). ME mucosal thickness at day 10 was 136.10 ± 17.26 μm and 33.76 ± 2.79 μm in the CS + OM and OM groups, respectively.

#### Expression of interleukin (IL)-1ß, IL-6, tumor necrosis factor (TNF)-α, vascular endothelial growth factor (VEGF), and hypoxia-inducible factor (HIF)-1α

**Figure 3:**
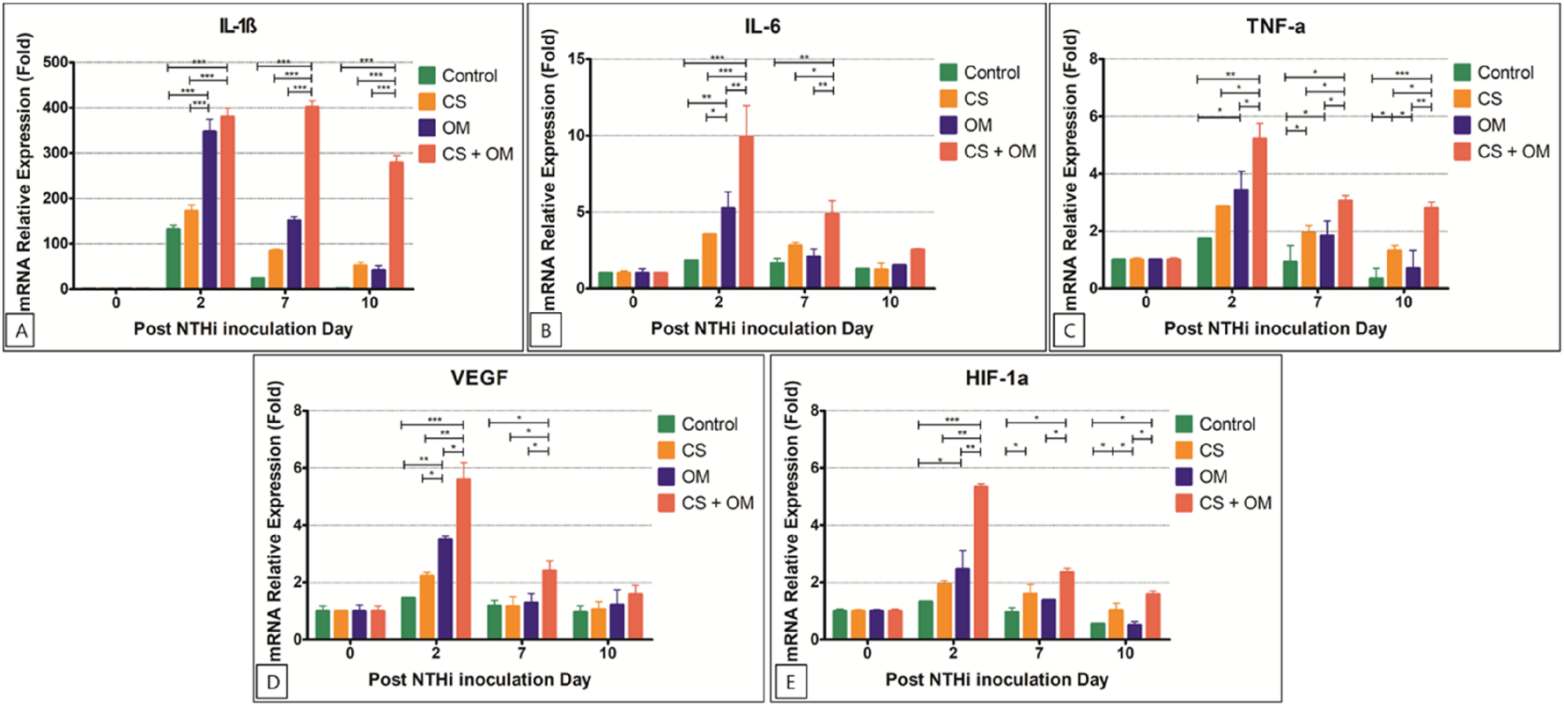
Expression of interleukin (IL)-1ß (**A**), IL-6 (**B**), tumor necrosis factor (TNF)-α (**C**), vascular endothelial growth factor (VEGF) (**D**), and hypoxia-inducible factor (HIF)-1α (**E**) in the middle ear mucosa at various time points after non-typeable *Haemophilus influenzae* (NTHi) inoculation.

**Figure 4:**
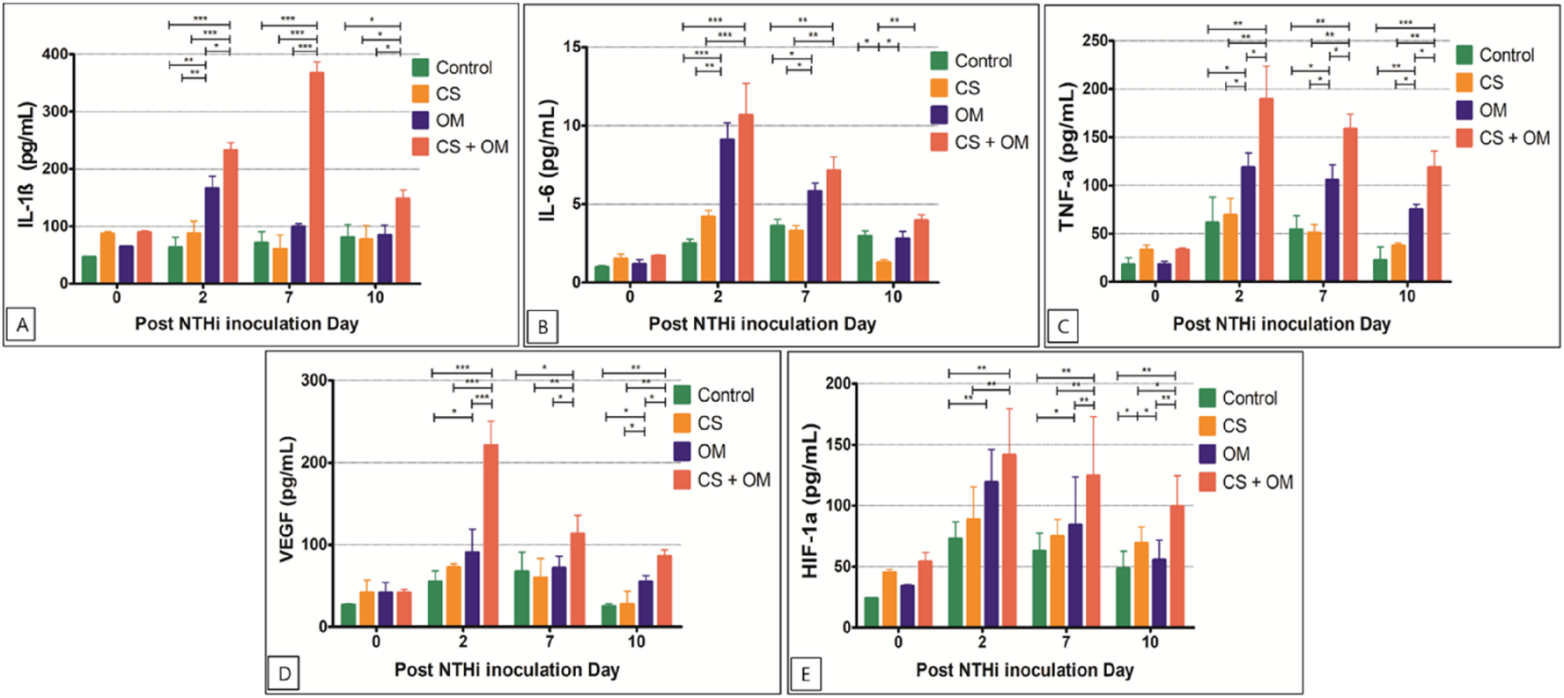
Cytokine profiles of interleukin (IL)-1ß (**A**), IL-6 (**B**), tumor necrosis factor (TNF)-α (**C**), vascular endothelial growth factor (VEGF) (**D**), and hypoxia-inducible factor (HIF)-1α (**E**) in the middle ear mucosa at various time points after non-typeable *Haemophilus influenzae* (NTHi) inoculation.

The mRNA expression of IL-1ß decreased more gradually until day 10 in the CS + OM group than in the other groups (Fig. 3). Higher IL-1ß mRNA expression was observed in the CS + OM group than in the OM group on days 7 (P < 0.001) and 10 (P < 0.001), while it decreased rapidly at day 7 in the OM group (Fig. 3A). The enzyme-linked immunosorbent assay (ELISA) results showed expression patterns similar to those of the quantitative polymerase chain reaction (qPCR) results (Fig. 4).

The mRNA expressions of IL-6, TNF-α, VEGF, and HIF-1α were more significantly upregulated in the CS + OM group than in the other groups at day 2 (Fig. 3). The mRNA expressions of IL-6 and VEGF showed no significant difference among the groups at day 10 (Fig. 3B,D). However, higher TNF-α expression was observed until 10 days after inoculation in the CS + OM group than in the control (P < 0.001) and OM groups (P = 0.008) (Fig. 3C). The mRNA expression of HIF-1α showed a pattern similar to that of TNF-α; however, no statistically significant differences were found between the CS + OM and CS groups at days 7 and 10 (Fig. 3E).

#### Histopathologic analysis and electron microscopic findings of the ET and ME

**Figure 5:**
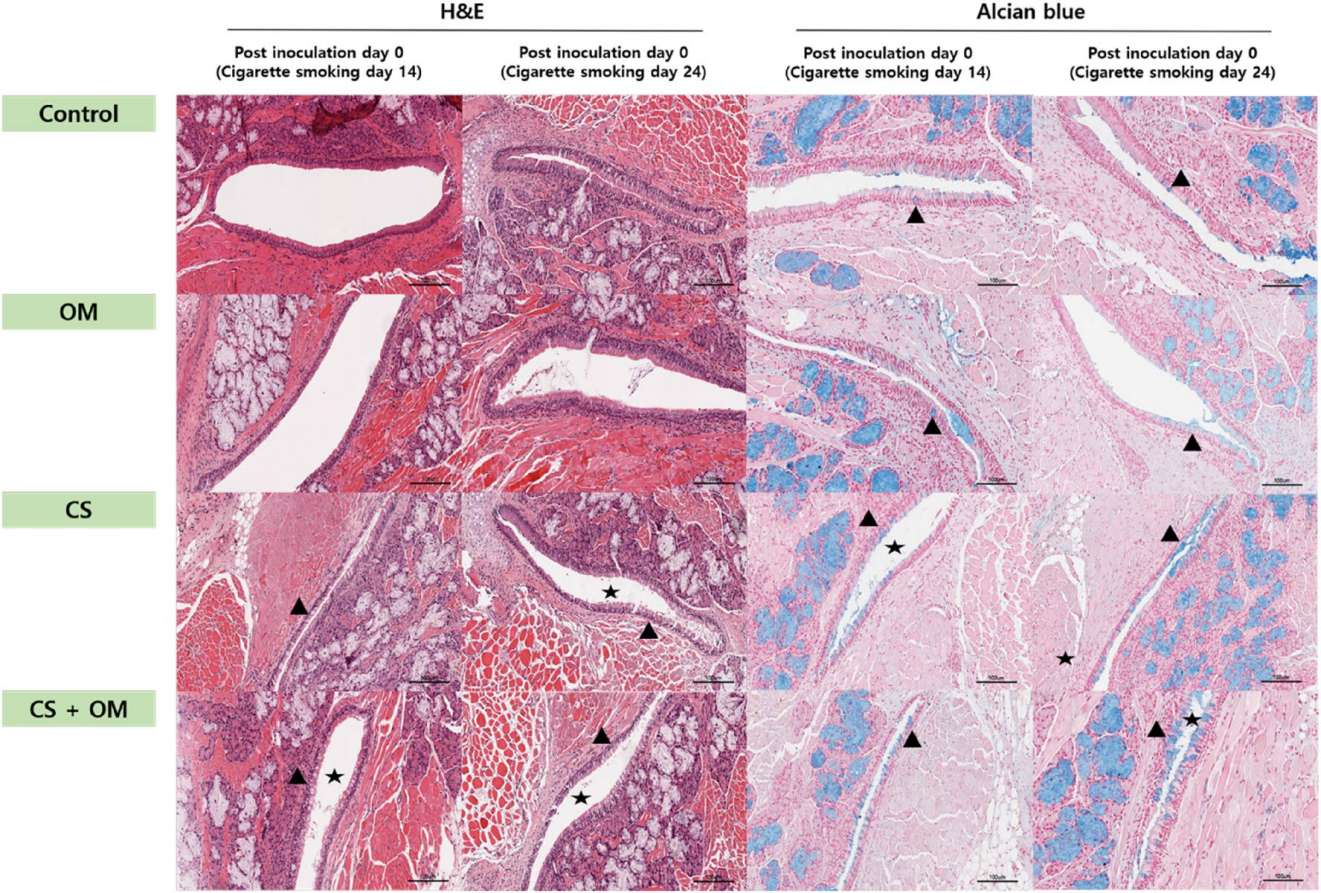
Light microscopic findings of the Eustachian tube at various time points. Left panels show sections stained using hematoxylin and eosin (×200 magnification); the right panels show sections stained using alcian blue (×200 magnification). The control and otitis media (OM) groups are not exposed to cigarette smoke (CS). At day 0 of inoculation, the CS and CS + OM groups have already been exposed to CS for 2 weeks. The loss of cilia (asterisk) and the number of goblet cells (arrowheads) are greater in the CS and CS + OM groups than in the control and OM groups.

The ETs of rats in the control and OM groups contained goblet cells and pseudostratified mucociliary respiratory-like epithelium (Fig. 5). After 2 weeks of CS exposure (day 0 after inoculation), the loss of cilia and squamous metaplasia were observed in 32% of the circumferential length of the ET lumen. Neutrophil infiltration was also observed in the subepithelial matrix. The numbers of goblet cells per high-power field (HPF) were 24.1 ± 6.8, 20.5 ± 5.1, 62.3 ± 15.8, and 56.0 ± 21.2 in the control, OM, CS, and CS + OM groups, respectively. The density of goblet cells in the CS and CS + OM groups was higher than that in the control and OM groups. After 24 days of CS exposure (day 10 after inoculation), the numbers of goblet cells were still higher and showed more dysmorphic changes in the CS and CS + OM groups than in the control and OM groups. The numbers of goblet cells per HPF were 31.2 ± 13.6, 54.5 ± 21.5, 69.1 ± 29.3, and 89.7 ± 34.7 in the control, OM, CS, and CS + OM groups, respectively. The number of goblet cells per HPF was significantly higher in the CS + OM group than in the OM group (P = 0.03). Moreover, destruction of the overlying epithelium was observed. However, no differences were observed between the control and CS groups at days 0 and 10.

**Figure 6:**
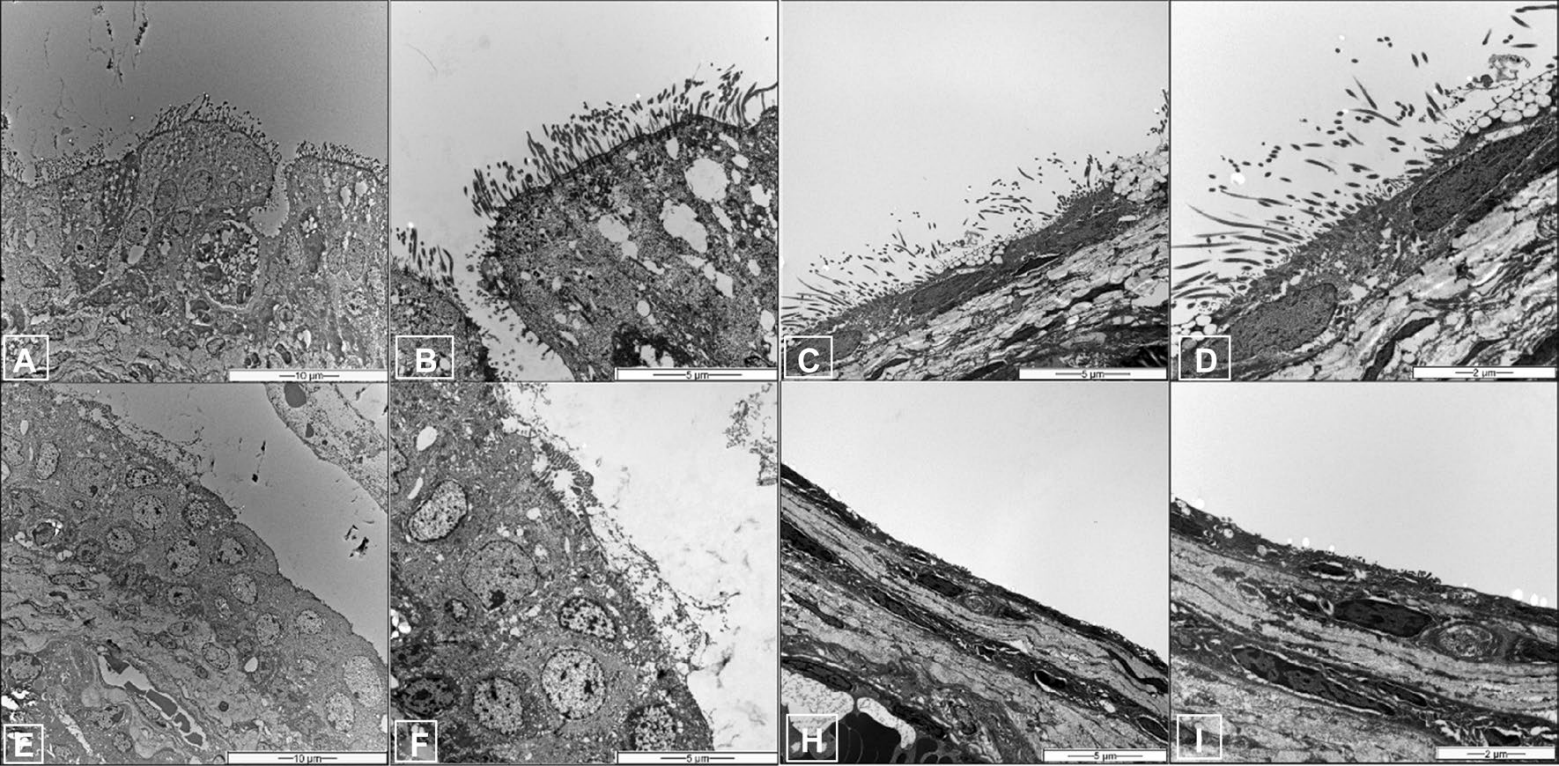
Ultrastructural findings of the Eustachian tube (**A**,**B**,**E**,**F**) and middle ear (**C**,**D**,**G**,**H**) in the control group. Normal cilia are observed in the Eustachian tube and middle ear. (**A**) ×2000 magnification. (**B**) ×6000 magnification. (**C**) ×5000 magnification. (**D**) ×10,000 magnification. Ultrastructural findings of the Eustachian tube and middle ear after 2 weeks of exposure to cigarette smoke. Severe loss of cilia and neutrophilic infiltration of the epithelium are observed. (**E**) ×2000 magnification. (**F**) ×5000 magnification. (**G**) ×5000 magnification. (**H**) ×10,000 magnification.

Electron microscopic analysis of the mucosa of the ET in the control group revealed goblet cells and pseudostratified mucociliary respiratory-like epithelium with cilia (Fig. 6A,B). The ultrastructural changes in the ET observed after 2 weeks of CS exposure included goblet cell proliferation and a severe loss of cilia in the CS and CS + OM groups at day 0 (Fig. 6E,F). Electron microscopic findings of the ME mucosa were similar to those of the ET (Fig. 6C,D,G,H).

### In vitro studies

#### Cigarette smoke condensate (CSC)-related cell viability of human ME epithelial cells (HMEECs)

**Figure 7:**
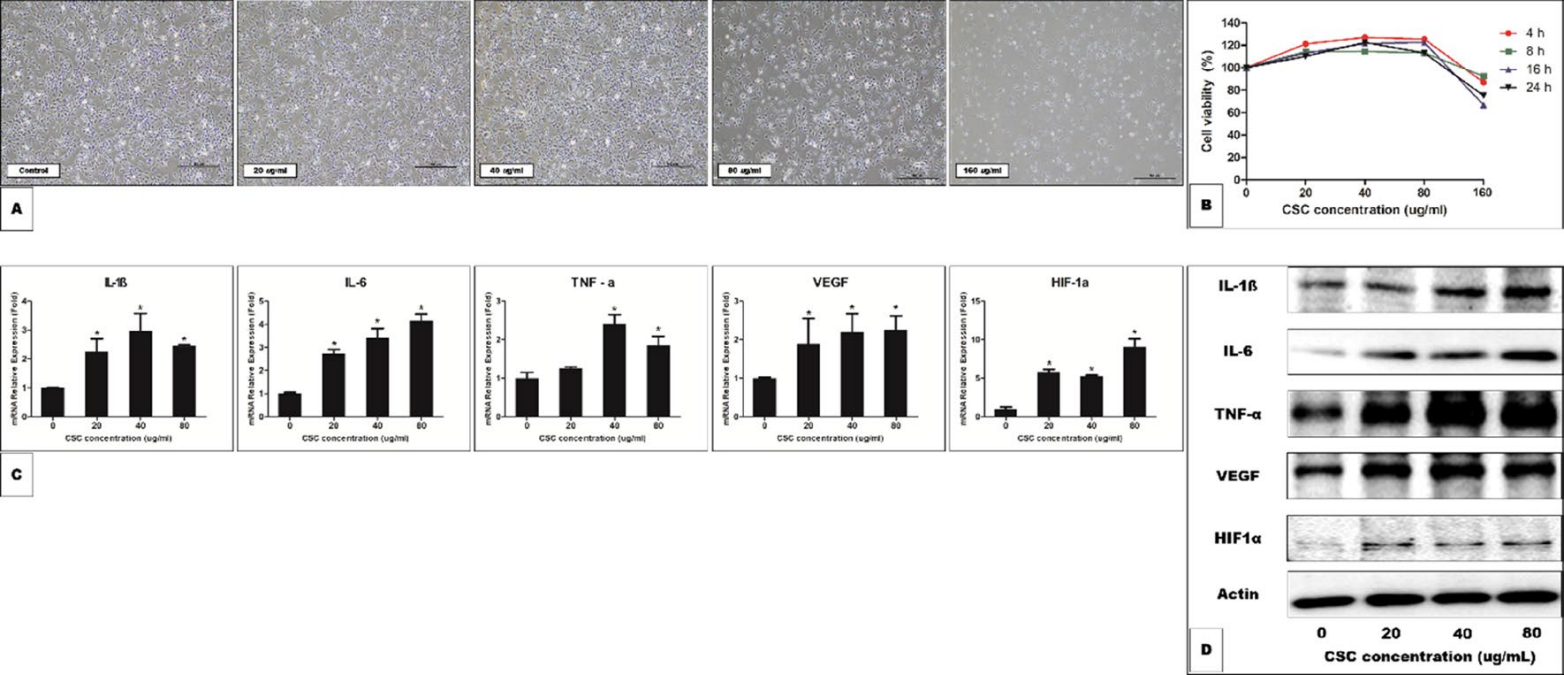
(**A**) Cell morphology of human middle ear epithelial cells (HMEECs) following exposure to cigarette smoke condensate (CSC) for 24 h. (**B**) The cell counting kit-8 assay reveals that cell viability decreases after the cells are exposed to 160 µg/mL CSC for 4, 8, 16, and 24 h. (**C**) Expressions of interleukin (IL)-1ß, IL-6, tumor necrosis factor (TNF)-α, vascular endothelial growth factor (VEGF), and hypoxia-inducible factor (HIF)-1α are upregulated by treatment with CSC. *P < 0.05. (**D**) The expression levels of IL-1ß, IL-6, TNF-α, VEGF, and HIF-1α are detected using western blotting.

The number of viable cells did not show a decrease with increasing time, but decreased as the CSC concentration increased above 160 µg/mL (Fig. 7A). Moreover, cell viability assays (Cell Counting Kit-8 [CCK-8]) showed that exposure to more than 160 µg/mL of CSC for 6, 12, and 24 h significantly decreased the viability of HMEECs when compared to that of the control cells (Fig. 7B).

#### CSC increases the expression of inflammatory cytokines (IL-1ß, IL-6, TNF-α), VEGF, and HIF-1α in HMEECs

To determine the effect of CSC on inducing inflammation in HMEECs, we evaluated the effect of CSC at concentrations of 20, 40, and 80 µg/mL on the upregulation of IL-1ß, IL-6, and TNF-α mRNA expression. The evaluation was not performed at concentrations above 160 µg/mL because of decreased cell viability. As shown by quantitative RT-PCR in Fig. 7C, the mRNA expression of IL-1ß and IL-6 in HMEECs was significantly increased by a 24-h stimulation with CSC at concentrations of 20, 40, and 80 µg/mL. TNF-α mRNA expression in HMEECs was significantly increased by a 24-h stimulation with CSC at concentrations 40 and 80 µg/mL. In subsequent experiments, the effects of CSC on VEGF and HIF-1α mRNA expression in HMEECs were evaluated. CSC increased VEGF and HIF-1α expression over a time course similar to that of the inflammatory cytokines, with maximal VEGF and HIF-1α mRNA levels being approximately two-fold (VEGF) and nine-fold (HIF-1α) higher than those of the controls by 24 h (Fig. 7C). The western blot results showed that the expression levels of IL-1ß, IL-6, TNF-α, VEGF, and HIF-1α increased with an increase in the CSC concentration (Fig. 7D). The full length gels and blots are included in the supplementary Fig. 1.

## Discussion

Smoking causes several pathologic changes and is a major predisposing factor in certain diseases^19^. The toxic effect of direct and passive smoke exposure on health has received considerable attention^20^. Direct smoke is defined as the mainstream smoke that the smoker inhales directly through the end of the cigarette. Passive smoke is defined as exposure to smoke by non-smoker. Further, mainstream smoke is defined as the smoke exhaled by the smoker, and sidestream smoke is defined as the smoke from burning cigarette^21^. Previous studies have documented that CS exposure induces histomorphological changes in the respiratory epithelium, including epithelial cell hyperplasia, metaplasia with keratinization, and ciliary loss with neutrophilic infiltration^20–23^. Moreover, several human studies have shown that CS causes hypertrophy of lymphatic tissues of the nasopharynx and adenoids, and eventually contributes to the onset of and delayed healing in OM^24–26^. In addition, CS has been proposed to lead to poor outcomes because of defective ME aeration secondary to the above effects^27^. Although the adverse effects of CS on the healthy mucosa have been documented in animal histological studies^17,18^, few histopathological studies have investigated the effects of CS on acute OM. Therefore, we investigated the effects of CS on acute OM, focusing on the period of OM recovery after bacterial infection and the proliferative response of the ME mucosa, which are the major components of OM pathology. In addition, we evaluated the effects of CS on the histopathological changes induced by acute OM in the ET mucosa.

All ears in the OM and CS + OM groups showed OM on day 2, and 60% of ears in the CS + OM group still had OM on day 10. ME mucosal thickness increased in all groups after PBS or NTHi inoculation, and the CS + OM group exhibited the greatest thickness. The OM group showed the peak thickness on day 2 but showed a steep decrease thereafter; however, the CS + OM group showed a gradual decrease until day 10. In the OM group, cytokine expression peaked on day 2 and decreased steeply thereafter. In the CS + OM group, cytokine expression increased and decreased gradually until day 10. Goblet cell proliferation and mucus secretion in the ET were more significant in the CS and CS + OM groups than in the other groups. In this study, ME mucosal thickness and the expression of proinflammatory cytokines (IL-1ß, IL-6, and TNF-α) increased in the PBS-injected control and CS groups, as well as in the OM and CS + OM groups, after inoculation. We believe that the surgical dissection performed to approach the bulla (i.e. the ME cavity) might have induced subsequent inflammation of the mucosa and soft tissue around the bulla opening site because the bony bulla wall might have been punctured while injecting PBS. However, clear differences were observed in the elevation of ME mucosal thickness and proinflammatory cytokine levels between the PBS-injected and NTHi-injected groups.

The inflammatory reaction was more intense and lasted longer in the CS and CS + OM groups than in the control and OM groups. Dubin et al. reported that CS exposure altered ET function in animals^27^. Our results also showed significantly elevated passive opening and closing pressures of the ET in rats exposed to CS than in the controls. Gryczyńska et al. reported that passive CS contributes to the onset and delay of healing in OM^26^. We observed goblet cell hyperplasia and increased staining of intracellular mucin in the ET of the CS and CS + OM groups as seen in an earlier study on the CS rat model^18^. Moreover, the histologic changes in the ET mucosa after CS exposure included the loss of cilia and squamous metaplasia^18^. In the current study, the CS group showed findings consistent with those of previous studies. These results imply that pre-existing and co-existing CS exposure that may mediate changes in the ET might be an important factor in determining the aggravation of inflammation and its duration. Although the pathomechanism underlying the relationship between acute OM and CS exposure remains unknown, previous studies have suggested that CS upregulates VEGF levels in the lower airways of the rat and human fetal lung, showing correlations with HIF-1α levels^28,29^. Our study showed similar findings indicating that CS exposure strongly enhanced VEGF and HIF-1α expression in the rat model of OM infected with NTHi. Nevertheless, further investigation is required to confirm this hypothesis.

Although our study demonstrated that CS affects the progression and subsequent recovery of acute bacterial OM, this study has a few limitations in terms of the interpretation of the results. This study focused on acute bacterial OM related to CS exposure. OM with effusion on CS exposure may initially be related to infection and may have different mechanisms. The results of this study cannot be applied to all cases of OM related to CS exposure. To elucidate OM development and recovery in subjects exposed to CS, further investigations on OM with effusion related to CS exposure are warranted.

The effect of CS on OM has received little attention, and few previous studies have investigated the effect of CS on the development, progression, and recovery of acute OM. To our knowledge, this is the first experimental study to assess the relationship between CS and bacterial OM. Our results showed a significant association between CS and bacterial OM and provided data supporting the hypothesis that CS could affect ME function through mucosal hypersecretion by goblet cells within the ET. A pathologic increase in goblet cell density and increased staining of intracellular mucin in the ET are associated with actual goblet cell proliferation as well as mucin synthesis, storage, and exocytosis^18^. CS could cause ET obstruction, dysfunction, and trapping of mucus and bacteria within the ME in acute OM^13^. We demonstrated that the pathological changes in the ME mucosa resulting from the induced acute bacterial OM were more severe and required a longer recovery period in the presence of comorbid CS exposure than in the absence of CS exposure. This indicates that CS affects the progression and subsequent recovery of acute bacterial OM. The inflammatory reaction to NTHi was also more intense and lasted longer in the CS + OM group than in the other groups. These findings suggested that because CS directly affects the ET and ME mucosa, bacterial OM can develop to a more severe stage and may resolve more slowly in the presence of CS exposure rather than in its absence.

## Methods

### Animals and study groups

**Figure 8:**
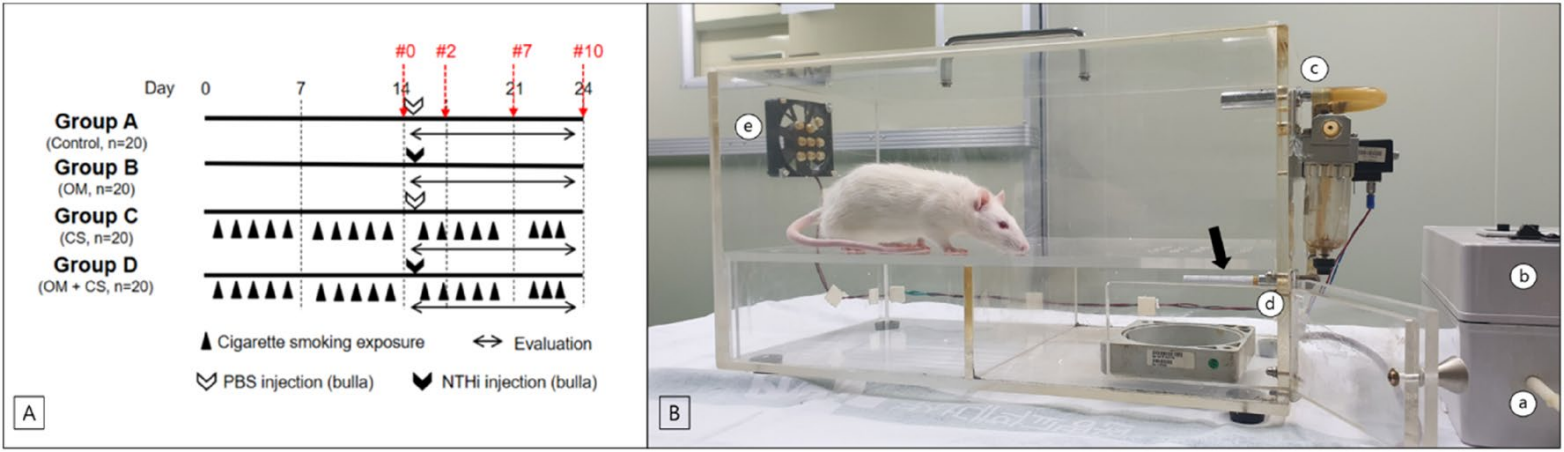
(**A**) Experimental protocol for generating the rat models of otitis media (OM), cigarette smoke (CS), and otitis media + cigarette smoke (CS + OM). (**B**) Experimental equipment used for CS exposure. The experimental animals are exposed to both mainstream and sidestream CS. a. Animal ventilator; b. interval controller; c. mainstream smoke; d. sidestream smoke; e. window; black arrow, cigarette.

In total, 80 male Sprague–Dawley rats (age, 7 weeks old; weight, 200–250 g) with normal MEs were studied. The breeding room was kept at a temperature of 22 °C, a humidity level of 40%, and a 12-h cycle of light and darkness. The 80 rats were randomly divided into four groups (n = 20 rats/group): control, OM, CS, and OM exposed to CS group (CS + OM) (Fig. 8A). The control and experimental groups were further subdivided into sets of 5 rats (10 ears), one for each of the four time points (0, 2, 7, and 10 days after inoculation, at which point the rats were sacrificed). The Institutional Animal Care and Use Committee (IACUC) of our hospital approved all experimental protocols for this study, and all procedures were carried out in accordance with the approved institutional guidelines of the IACUC (ID no. PUNH-2020-160) and conducted in accordance with the National Institutes of Health Guide for the Care and Use of Laboratory Animals, the ARRIVE guidelines, and under consideration of the 3R-principle.

### Experimental OM rat model

Bacterial OM was induced in the OM and CS + OM groups. NTHi 3655 stored at − 80 °C was streaked on chocolate agar plates (BD cat # 21169, New Jersey, USA). The batch was incubated overnight at 37 °C in a humid atmosphere containing 5% CO_2_. Colonies were collected and cultured in 25 mL of brain heart infusion broth (Teknova, B9500, California, USA) supplemented with 1 mL of Fildes Enrichment (Remel, R45037, California, USA). After overnight incubation at 37 °C, the mixture was centrifuged to obtain a pellet. A bacterial suspension of 1 × 10^5^ NTHi cells/mL was used in the experiment^4,9,30^. All the rats with normal tympanic membranes were anesthetized via intraperitoneal injection of an anesthetic mixture of Rompun (Bayer, Leverkusen, Germany) and Zoletil (Virbac, Texas, USA) at a dose of 1 mL/kg. Lidocaine-hydrochloric acid with adrenaline (dilution ratio, 1:100,000; Huons, Seongnam-si, Republic of Korea) was also injected before incision for inducing local anesthesia and homeostasis. The operation was performed under surgical microscopic guidance. Briefly, while the rat was held in the supine position, a vertical 3-cm incision was made on the anterior neck. The soft tissue was dissected and both bullae were exposed. A 25-gauge needle was used to pierce a hole at the bulla tip, and 0.05 mL of the bacterial suspension was injected into the hole^4,9,30^. The same anesthetic solution was used on days 0, 2, 7, and 10 after inoculation when acquiring photographs of the tympanic membrane to check the progression of OM. Prior to sacrificing the rats, the ME effusion and tympanic membrane statuses were confirmed using a model AM4113EUT (R4) portable digital microscope (USB) (Dino-Lite, New Taipei City, Taiwan).

### Exposure to CS

The CS exposure protocol has been previously described^17,18,31^. Briefly, a custom smoking chamber (50 × 25 × 27 cm) was used (Fig. 8B). The rats in the CS and CS + OM groups were placed within the chamber on an acrylic plate above the cigarette. The chamber consists of two interconnected spaces divided above and below by an acrylic plate. Lighted cigarettes are placed in the space below the acrylic plate. This space has an external pump connected to it through which air is pumped to form a stream that leads the smoke into the space above the acrylic plate, where the animals are located. The space above the acrylic plate has a window that acts as a vent for the pumped air.

One exposure in the chamber was defined as an exposure to one regular-sized filtered cigarette (tar, 12 mg; nicotine, 1.0 mg; Marlboro-Red, Philip Morris International, Neuchâtel, Swiss) every 12 min for a total of 1 h (i.e. 5 cigarettes) and 5 days/week. Smoke inhalation was initiated by burning each cigarette at a rate of 1 puff/min (puff duration, 2 s; total, 12 puffs/cigarette) to a final butt length of 23 mm. The carbon monoxide concentration in the chamber ranged from 335.35 to 341.27 ppm. All 5 rats in a group were placed in the chamber simultaneously. The cigarette was placed at the end of a tube that was attached to the animal ventilator. The rats were exposed to mainstream smoke when the smoke was pulled through the ventilator and pushed into the chamber, as well as to sidestream smoke when the smoking gas was released from the burning end of a cigarette^17,18,31^. The rats were sacrificed 1 h after the last exposure.

### Histopathological analysis

Prior to sacrificing the rats, the ME effusion and tympanic membrane statuses were confirmed. Tympanic bullae were harvested and fixed in 4% paraformaldehyde (P2031, Biosesang, Seongnam-si, Republic of Korea) for 24 h at 4 °C and rinsed with PBS and decalcified in 10% ethylenediaminetetraacetic acid (EDTA; 17385S0401, Junsei, Tokyo, Japan) solution (pH 7.8) for 3 weeks. The softened bullae were dehydrated through a 70%, 80%, 90%, 95%, and 100% alcohol and xylene gradient, and embedded in paraffin. The paraffin-embedded bullae were sectioned into 4-µm longitudinal sections for staining. The sectioned bullae were deparaffinized, rehydrated, and stained with hematoxylin and eosin (H&E) (Sigma Aldrich, St. Louis, USA) to visualize the ME mucosa. Thereafter, ME mucosal thickness was measured from three selected areas to obtain an overall average thickness, including one area where an imaginary line was drawn from the cochlea to the opposite bulla wall, and two areas where a line perpendicular to the imaginary line was drawn to meet the bulla walls by using a Leica Aperio CS2 with Aperio ImageScope and an image analysis system (Leica Microsystems Imaging Solutions Ltd., Cambridge, UK) (Suppl. Figure 2).

The ET was separated from the ME via a scalpel incision, ensuring the preservation of the tympanic orifice in the ET. Tissue sections were embedded in paraffin and sectioned into 4-µm slices perpendicular to the longitudinal axis of the ET. The middle portion of the tube was stained using H&E and alcian blue. An examiner blinded to the groups counted the number of goblet cells under HPFs (10 × 40) and recorded the mean numbers in each group.

### qPCR and ELISA using ME mucosal specimens

Total RNA was extracted from the ME mucosa of the experimental and control rats by using the TriZol reagent (Invitrogen, Carlsbad, USA). cDNA was synthesized using the SmartGene compact cDNA Synthesis kit (SMART GENE, Daejeon, Republic of Korea). qPCR was performed using the SmartGene Sybr Green Q-PCR Master Mix with Low Rox (SMART GENE) according to the manufacturer’s instructions twice to determine the expression of IL-1ß, IL-6, TNF-α, VEGF, and HIF-1α; glyceraldehyde 3-phosphate dehydrogenase values were used to normalize gene expression (Suppl. Table 1).

ELISA kits were used to determine the expressions of IL-6, TNF-α, and VEGF (CUSABIO, Hubei, China) and IL-1ß and HIF-1α (MyBioSource, San Diego, USA). After measuring the optical density at 450 nm, the levels of IL-1ß, IL-6, TNF-α, VEGF, and HIF-1α were determined via interpolation from a standard curve, and all data were expressed as nanograms per milliliter.

### Transmission electron microscopic findings of the ET

Tissues were harvested in the same manner as described above. Harvested tissues were prefixed with 2.5% glutaraldehyde solution (4 °C; phosphate buffer, pH 7.2) and decalcified with EDTA for 6 weeks and post-fixed with 1% osmium tetroxide (4 °C; phosphate buffer, pH 7.2). The tissues were washed with the same buffer and dehydrated using a graded alcohol series and subsequently blocked and fixed with Epon 812 mixture solution. Thereafter, the tissues were cut into 1-μm-thick sections and dyed with 1% toluidine blue. Ultrathin sections (50– 70 nm) were obtained using an ultramicrotome (EM UC7, Leica, Wetzlar, Germany), double-stained with uranyl acetate and lead citrate, and observed under a transmission electron microscope (JEM 1200EX-II, JEOL, Tokyo, Japan).

### In vitro studies

Methods related to in vitro studies are described in supplementary methods^32–34^. The contents of CSC are listed in supplementary Table 2. Forward and reverse oligonucleotides for PCR amplification of IL-1ß, IL-6, TNF-α, VEGF, and HIF-1α are shown in supplementary Table 3.

### Statistical analysis

All data are presented as the mean ± standard error of the mean. Statistical analysis was performed using IBM SPSS Statistics for Windows, Version 22.0 (IBM Corp., Armonk, USA). A P-value < 0.05 was considered statistically significant. The occurrence of OM at each time point among the groups was compared using Fisher’s exact test. ME mucosal thickness was analyzed using one-way analysis of variance with post-hoc comparisons among groups. Kruskal–Wallis tests were used to compare intergroup differences in the expression of the proinflammatory cytokines (IL-1ß, IL-6, and TNF-α), and Mann–Whitney U tests were used to test for differences between two groups.

## Supplementary Information

Supplementary Information.

## Data Availability

The datasets generated during and/or analyzed during the current study are available from the corresponding author on reasonable request.
