# Supplementary material for: Effects of cigarette smoke on Haemophilus influenzae-induced otitis media in a rat model
Source: Sci Rep. 2021 Oct 5;11:19729. doi: 10.1038/s41598-021-99367-w (PMC8492685; doi:10.1038/s41598-021-99367-w)
Supplement: Supplementary file 1 — Supplementary Information. [file 41598_2021_99367_MOESM1_ESM.docx]

**Supplementary Information**

**Effects of Cigarette Smoke on *Haemophilus influenzae*-Induced Otitis Media in a Rat Model**

Sung-Won Choi^1^, Sunmi Choi^1^, Eun-Jin Kang^1^, Hyun Min Lee^2^, Se-Joon Oh^1^, Il-Woo Lee^2^, Hwan Ho Lee^3^, Soo-Keun Kong^1,*^

**Supplementary Methods**

- 1. **CSC preparation**

CSC was kindly provided by Prof. Eun Sang Choe, Department of Biological Sciences, Pusan National University^32^. All samples were conditioned to routine analytical smoking machine definitions and standard conditions according to the standards of the International Organization for Standardization. CSC was prepared using a Cambridge filter pad (44 mm, Whatman, Maidstone, UK) and then dissolved with 1% dimethyl sulfoxide (DMSO) to a total particulate matter concentration of 25.7 mg/mL. CSC was filtered using 0.45-μm polytetrafluoroethylene sterile filters and kept at -80°C until used. This experiment was conducted on the basis of the concentration of whole CSC. The contents of CSC are listed in supplementary Table 2.

- 1. **HMEEC culture**

HMEECs were kindly provided by Prof. Moo Kyun Park, Department of Otorhinolaryngology-Head and Neck Surgery, Seoul National University Hospital^33,34^. HMEECs were cultured in a mixture of Dulbecco’s modified Eagle’s medium (LM001-05, Welgene, Korea) supplemented with BEGM SingleQuots (CC-4175, Lonza, Switzerland) and 10% fetal bovine serum (Gibco Life Technologies, New York, USA) and 1% penicillin-streptomycin (Gibco Life Technologies). Cells were maintained in a humidified incubator at 37°C containing 95% air and 5% CO_2_. To study the effects of CSC, the cells were grown to 1 × 10^5^ cells/well in six-well culture plates at 37°C. After 24 h, the cells were treated with 20, 40, 80, and 160 µg/mL CSC, and the control group cells were treated with 0.1% DMSO (D2438-50ML, Sigma).

- 1. **Cell viability**

Cell viability was measured by using the CCK-8 Assay (Dojindo Laboratories, Kumamoto, Japan). The cells were seeded in 96-well plates at 1 × 10^4^ cells/well. After 24 h, the cultured cells were stimulated with CSC at concentrations of 20, 40, 80, and 160 µg/mL for 4, 8, 16, and 24 h. Thereafter, 10 µL CCK-8 reagent was added to each well, and the plates were incubated in the dark for 1 h at 37°C. The absorbance was measured using a Hybrid Multi-Mode Microplate Reader (Synergy H1, BioTek, Winooski, USA) at a wavelength of 450 nm.

- 1. **Quantitative real-time reverse transcription PCR using HMEECs**

Total RNA was prepared from the cells by using TriZol reagent, with some changes according to the manufacturer’s instructions. HMEECs were seeded in 6-well plates, with each well containing 5 × 10^3^ cells. After 24 h, the incubated cells were treated with CSC at concentrations of 20, 40, and 80 µg/mL. Thereafter, 200 µL of TriZol reagent was added to each well, and the cells were collected in an eppendorf tube by concentration using a scraper. Then, 120 µL of chloroform was added and incubated at room temperature for 10 min. Centrifugation was performed at 13,000 rpm for 15 min at 4°C, and the collected supernatant alone was transferred to a new eppendorf tube. Then, isopropanol was added and the sample was incubated at room temperature for 15 min. This was followed by another centrifugation at 13,000 rpm for 10 min at 4°C. The supernatant was carefully discarded, and the pellet was washed in 75% ethanol. After removing the leftover ethanol, the RNA pellet was completely dried and diluted in diethylpyrocarbonate-treated water. The extracted RNA was used to synthesized cDNA using a compact cDNA synthesis kit (SG-cDNAC100, SMART GENE). Quantitative real-time PCR was performed using SYBR Green Q-PCR Master Mix with Low Rox (SG-SYBR-ROXL, SMART GENE). The reaction mixtures were incubated at 95°C for 5 min to activate them, followed by amplification for 40 cycles (one cycle: 15 s at 95°C, 30 s at 60°C) using AB7500 Fast Real-time PCR (Thermo Fisher Scientific, Waltham, USA). All reactions were performed in triplicates. Forward and reverse oligonucleotides for PCR amplification of IL-1ß, IL-6, TNF-α, VEGF, and HIF-1α are shown in supplementary Table 3.

- 1. **Western blotting**

Total protein was extracted from the CSC-treated (0, 20, 40 or 80 µg/mL for 24 h) cultured cells using ProEXTM CETi Lysis Buffer (Translab, TLP-121CTEi, Korea), and incubated with lysis buffer on ice for 30 min and centrifuged at 13,000 rpm for 15 min at 4°C. After transferring the supernatant to a new 1.5-mL Eppendorf tube, the protein concentration was determined using the BCA protein assay kit (23227, Thermo Fisher Scientific). Protein samples were mixed with 5× loading dye (TLP-102.1, Translab, Korea) and heated to 100℃ for 5 min and loaded on 8–10% sodium dodecyl sulphate-polyacrylamide gel and electrophoresed. The products were then transferred to a polyvinylidene difluoride membrane (Millipore, Schwalbach, Germany) and blocked using a blocking buffer (TLP-115.1G, Translab, Korea) at room temperature for 1 h with shaking, and thereafter incubated at 4°C overnight with the primary antibodies for IL-1ß (SC-52012, 1:500), IL-6 (SC-28343, 1:250), TNFα (SC-52746, 1:500), VEGF (SC-7269, 1:500), HIF-1α (SC-13515, 1:250), and actin-HRP (SC-47778, 1:1000, Santa Cruz Biotechnology, Dallas USA). After washing three times with 1× Tris-buffered saline with 0.1% Tween 20 (TBST) for 10 min, the HRP-conjugated secondary antibodies were added and incubated at room temperature for 1 h. The membranes were rinsed with 1× TBST three more times, and the blot was visualized using EZ-Western Lumi Femto kit (#DG-WF100, DoGen, Korea) and imaged using AE-9150 Ez-Capture 2 (ATTO, Korea).


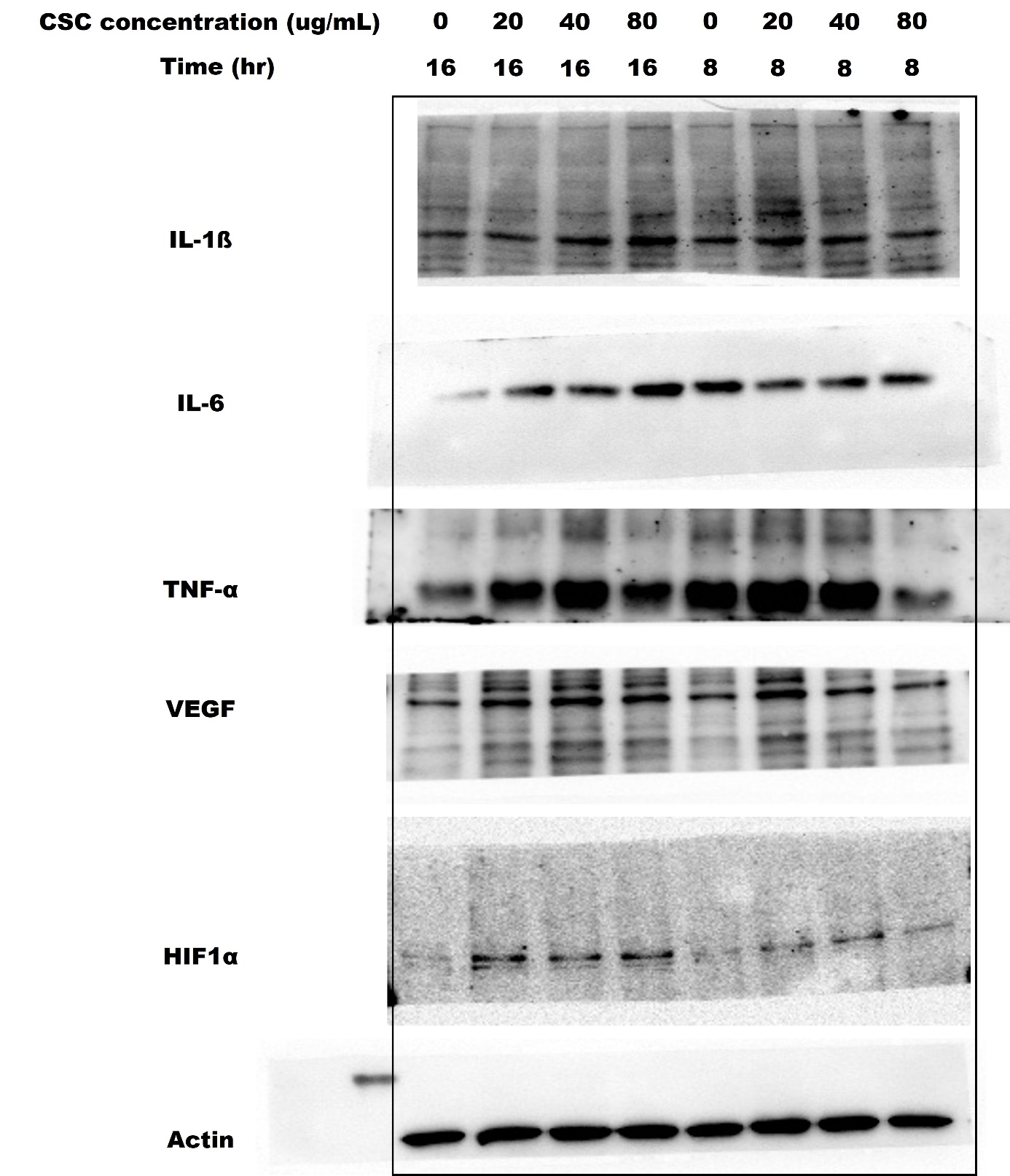


**Supplementary Fig. 1.** Original blots of Figure 7D.


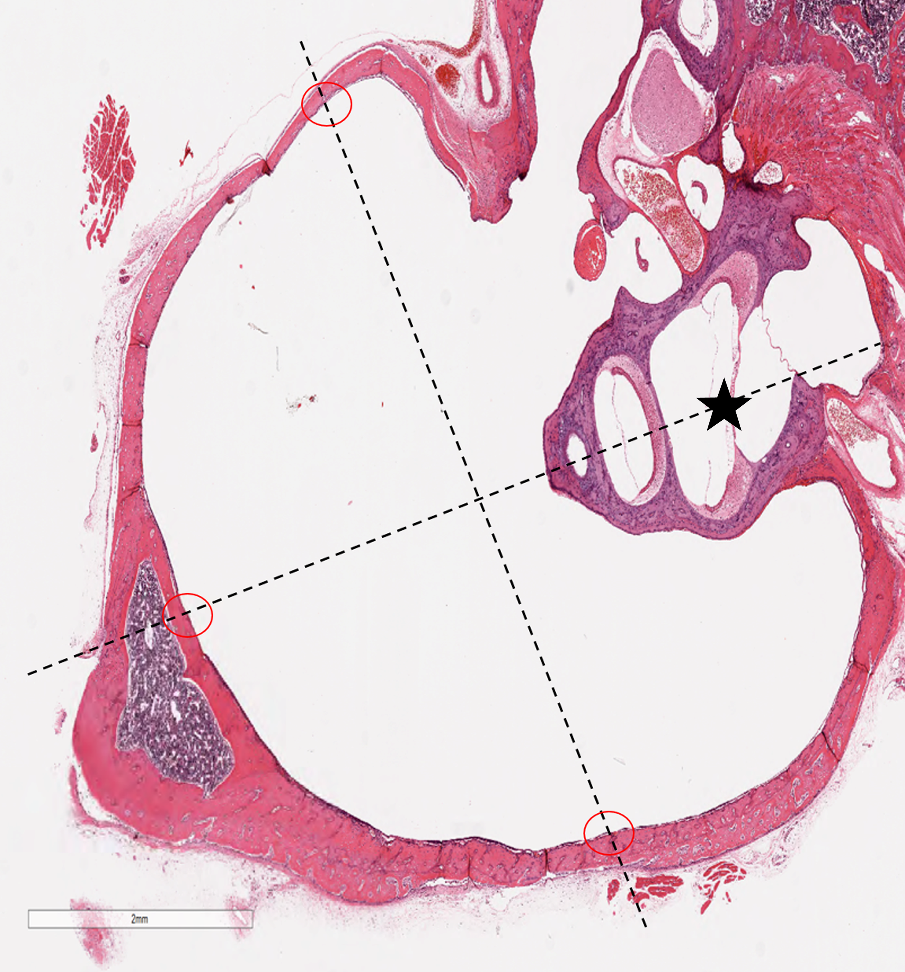


**Supplementary Fig. 2.** Light microscopic measurements of the middle ear (ME) mucosal thickness in the control group (× 20 magnification). ME mucosal thickness is measured from three selected areas (red circles) to obtain an overall average thickness, including one area where an imaginary line is drawn from the cochlea (asterisk) to the opposite bulla wall, and two areas where a line perpendicular to the imaginary line is drawn to meet the bulla walls.

**Supplementary Table 1. Polymerase chain reaction primer sequences for rats.**

|  | Sequence (5' - 3') | | Product size (bp) |
| --- | --- | --- | --- |
| IL-1ß | Forward | GCAATGGTCGGGACATAGTTGA | 158 |
|  | Reverse | AGACCTGACTTGGCAGAGGA |  |
| IL-6 | Forward | ACCCCAACTTCCAATGCTCT | 135 |
|  | Reverse | GGTTTGCCGAGTAGACCTCA |  |
| TNF-a | Forward | ACCACGCTCTTCTGTCTACTG | 170 |
|  | Reverse | TGCTTGGTGGTTTGCTACGAC |  |
| VEGF | Forward | TTCAACGGACTCATCAGCCA | 162 |
|  | Reverse | AGGGAGTGAAGGAGCAACCT |  |
| HIF-1a | Forward | ATTTAGAGGCCTGGCTACAGT | 283 |
|  | Reverse | GGAGCTGTGAATGTGCTGTGA |  |
| GAPDH | Forward | GATGGTGAAGGTCGGTGTGA | 163 |
|  | Reverse | GAACTTGCCGTGGGTAGAG |  |

*IL: interleukin, TNF: tumor necrosis factor, VEGF: vascular endothelial growth factor, HIF: hypoxia-inducible factor, GAPDH: glyceraldehyde 3-phosphate dehydrogenase*

**Supplementary Table 2. Total particulate matter (TPM) and nicotine concentrations^32^.**

| Sample code | Total particulate matter | | Nicotine | | Formaldehyde | | Acetaldehyde | |
| --- | --- | --- | --- | --- | --- | --- | --- | --- |
|  | (mg/cigarette) | (mg/mL) in WCSC | (mg/cigarette) | (mg/mL) in WCSC | (µg/cigarette) | (µg/mL) in WCSC | (mg/cigarette) | (µg/mL) in WCSC |
| 3R4F | 16.1 | 25.7 | 1.08 | 1.73 | 10.6 | 16.9 | 53.6 | 85.7 |

**Supplementary Table 3. Polymerase chain reaction primer sequences for the HMEECs.**

|  | Sequence (5' - 3') | | Product size (bp) |
| --- | --- | --- | --- |
| IL-1ß | Forward | CATTGCTCAAGTGTCTGAAGC | 238 |
|  | Reverse | CATGGCCACAACAACTGACG |  |
| IL-6 | Forward | ACTCACCTCTTCAGAACGAATTG | 149 |
|  | Reverse | CCATCTTTGGSSGGTTCAGGTTG |  |
| TNF-a | Forward | CCCATGTAGCAAACCT | 132 |
|  | Reverse | TGAGGTACAGGCCCTCTGAT |  |
| VEGF | Forward | TCCACCATGCCSSGTGGTC | 128 |
|  | Reverse | GTCCACCAGGGTCTCGSTTG |  |
| HIF-1a | Forward | GAAAGCGCAAGTCCTCAAAG | 166 |
|  | Reverse | TGGGTGGAGSTGGAGATGC |  |
| GAPDH | Forward | GAGTCAACGGATTTGGTCGT | 238 |
|  | Reverse | TTGATTTTGGAGGGATCTCG |  |

*IL: interleukin, TNF: tumor necrosis factor, VEGF: vascular endothelial growth factor, HIF: hypoxia-inducible factor, GAPDH: glyceraldehyde 3-phosphate dehydrogenase*
